# Fatigue across different chronic kidney disease populations: experiences and needs of patients

**DOI:** 10.1093/ckj/sfaf118

**Published:** 2025-04-18

**Authors:** Eline Schade van Westrum, Ellen K Hoogeveen, Birit F P Broekman, Carl E H Siegert, Dirry Keurhorst, Coby Annema, Marc H Hemmelder, Willem Jan W Bos, Friedo W Dekker, Yvette Meuleman, the PRO-GUIDE study group, Eline Schade van Westrum, Friedo Dekker, Marc Hemmelder, Birit Broekman, Carl Siegert, Alferso Abrahams, Ellen Hoogeveen, Coby Annema, Karen Prantl, Wanda Konijn, Marc ten Dam, Mandy Smink, Mirjam Splinter, Dirry Keurhorst, Theodôr Vogels, Willem Jan Bos, Frans van Ittersum, Yvette Meuleman

**Affiliations:** Department of Clinical Epidemiology, Leiden University Medical Center, Leiden, The Netherlands; Department of Clinical Epidemiology, Leiden University Medical Center, Leiden, The Netherlands; Department of Internal Medicine, Nephrology, Leiden University Medical Center, Leiden, The Netherlands; Department of Nephrology, Jeroen Bosch Hospital, Den Bosch, The Netherlands; Department of Psychiatry, OLVG and Amsterdam UMC, Amsterdam Public Health Institute, Vrije Universiteit, Amsterdam, The Netherlands; Department of Nephrology, OLVG Hospital, Amsterdam, The Netherlands; Department of Social Work, Flevoziekenhuis, Almere, The Netherlands; Department of Health Sciences, Section of Nursing Science, University Medical Center Groningen, University of Groningen, Groningen, The Netherlands; Department of Internal Medicine, Erasmus Medical Center, Rotterdam, The Netherlands; Department of Internal Medicine, Nephrology, Leiden University Medical Center, Leiden, The Netherlands; Department of Internal Medicine, St. Antonius Hospital, Nieuwegein, The Netherlands; Department of Clinical Epidemiology, Leiden University Medical Center, Leiden, The Netherlands; Department of Clinical Epidemiology, Leiden University Medical Center, Leiden, The Netherlands

**Keywords:** chronic kidney disease, fatigue, patient-centred care, patient-reported outcomes, symptom burden

## Abstract

**Background:**

Fatigue is a common symptom of chronic kidney disease (CKD). Predominantly qualitative research among dialysis patients has contributed to our knowledge about CKD patients’ fatigue experiences and perceptions. This nationwide survey study aimed to explore in different CKD populations: (i) patients’ experienced fatigue burden, its impact on daily life, and presumed causes of fatigue; and (ii) patients’ experiences and needs regarding support, discussion, and treatment of fatigue.

**Methods:**

A survey assessing patients’ fatigue experiences and needs was constructed in co-creation with the Dutch Kidney Patients Association. Descriptive statistics were used to summarize results and stratified by CKD populations [CKD without kidney replacement therapy (KRT), receiving dialysis, after kidney transplantation (KTx)], gender, and age.

**Results:**

A high fatigue burden was found across all CKD populations (*n* = 414;144 CKD without KRT/39 dialysis/231 KTx): fatigue was often experienced (94.7%), present for >6 months (90.3%), in the top three most burdensome symptoms (86.3%), and presumed causes were multifactorial. Younger patients were limited in more life domains than elderly. Some patients (32.1%) never or rarely discussed fatigue with their physician, did not receive treatment (67.8%), or felt the advice/treatment(s) insufficiently managed their fatigue (58.6%). More women and 18–50-year-old patients reported insufficient social support. Patients desired acknowledgement and more information about treatments and coping strategies for (consequences of) fatigue.

**Conclusion:**

High fatigue burdens and insufficient support are experienced across all CKD populations, gender, and age groups. To address patients’ unfulfilled needs, it is important to structurally measure and discuss fatigue in routine nephrology care, strengthen social support, and provide patient-centred multidisciplinary symptom management.

KEY LEARNING POINTS **What was known:**

- Fatigue is a common symptom among patients with CKD.
- Predominantly qualitative research provides insight into dialysis patients’ experiences with and perspectives on fatigue, and into healthcare professionals’ perceived difficulties addressing fatigue in routine dialysis care.
- Contradictory evidence exists on how symptom experiences differ depending on patient characteristics (e.g. gender and age).

**This study adds:**

- Fatigue is prevalent, persistent, burdensome, and impactful across different CKD populations, gender, and age groups.
- Many patients do not discuss fatigue with their physician and receive insufficient information, (social) support, and treatment, resulting in unfulfilled needs.
- Presumed causes for fatigue are multifactorial and patients consider both pharmacological and nonpharmacological interventions as essential.

**Potential impact:**

- A better understanding of patients’ perceptions, experiences, and (support) needs regarding fatigue may help to inform future patient-centred interventions across different CKD populations.
- The high symptom burden, impact, and unmet needs underline the importance of PROMs implementation to enable structural measurement, discussion, and multidisciplinary symptom management of fatigue in routine nephrology care.

## INTRODUCTION

Throughout different stages of chronic kidney disease (CKD), patients experience multiple physical and emotional symptoms, of which fatigue is considered one of the most common symptoms [1]. The aetiology of fatigue remains unclear but it is presumably caused by (interactions between) biological, psychological, and social factors [2, 3]. Fatigue can profoundly impact patients’ daily lives [2, 4] and restrict their daily activities [5]. Until now, predominantly qualitative research among dialysis patients contributed to our knowledge about CKD patients’ experiences with and perspectives on fatigue [6–8].

A systematic review showed that patients across different CKD populations experienced fatigue: ∼70% of patients with CKD without kidney replacement therapy (KRT, hereafter called ‘patients with CKD’) and receiving dialysis, and ∼48% of patients after kidney transplantation (KTx) suffered from fatigue [1]. There are also indications that symptom experiences may differ depending on patient characteristics. For instance, elderly patients, whether they received KRT or not, experienced more fatigue [5]. Current evidence on gender differences is contradictory: some studies found that fatigue is experienced more frequently and severely by women than men receiving dialysis, while others found no differences [9]. Therefore, more research is needed to explore differences in fatigue experiences between different CKD, gender, and age groups.

Despite the high prevalence and burden of fatigue among patients in different CKD populations, it often remains underrecognized in routine nephrology care [10–12]. Possible explanations include patients’ discomfort in sharing their symptoms and needs with healthcare professionals, healthcare professionals’ struggle to identify the full spectrum and severity of patients’ symptoms and needs [13], and the perceived lack of opportunities to treat fatigue [6]. Currently, some attention is paid to healthcare professionals’ perspectives on addressing fatigue in routine dialysis care [6], but knowledge about patients’ experiences and (support) needs is lacking.

Therefore, this study aims to explore (i) patients’ experienced fatigue burden, its impact on daily life, and presumed causes of fatigue; and (ii) patients’ experiences and needs regarding support, discussion, and treatment of fatigue for the total population as well as the CKD populations, gender, and age groups separately.

## MATERIALS AND METHODS

### Study design and population

A nationwide cross-sectional web-based survey study was conducted in The Netherlands in co-creation with the national Dutch Kidney Patients Association (i.e. NVN). Patients in all stages of CKD with access to an internet-connected electronic device and sufficient knowledge of the Dutch language could participate between 10 January 2023 and 18 January 2024. To ensure a large and diverse sample representing the CKD population, the survey was distributed via the NVN's online panel, their members’ digital bimonthly newsletter and Kidney Magazine, social media, and the website nieren.nl (platform accessible for patients with CKD). We aimed to include at least 291 patients (see Item S1 in the Supplementary Data for the sample size calculation).

Completed and anonymous data was obtained from the NVN. All participants gave informed consent to collect and use the survey data for this scientific research. The study is not subject to the Medical Research Involving Human Subjects Act, but approval was obtained from the Scientific Committee of the Clinical Epidemiology Department at Leiden University Medical Center. The Checklist for Reporting of Survey Studies (CROSS) was used to report this study (Item S2) [14].

### Survey and data collection

The survey was developed by the NVN in close collaboration with an expert research team (e.g. health psychologist, nephrologist, and clinical epidemiologist) and was based on literature and existing surveys (e.g. Dialysis Symptom Index [15]). It was reviewed and tested by staff and volunteers from the national and local (Hollands Midden) Kidney Patients Associations, and feedback was incorporated. The online Spidox tool was used to create and conduct the survey.

The survey consisted of 21 closed-ended questions (including eleven options to provide alternative answers and/or explain answers) and six open-ended questions (Item S3). The following aspects were assessed: (i) experiences and perceptions of fatigue: symptom frequency in the past month, symptom duration (present for >6 months), symptom burden (severity score, whether in the top three most burdensome symptoms), how participants noticed fatigue [on a physical level (e.g. less strength), mental level (e.g. problems concentrating), emotional level (e.g. getting easily upset), and/or social level (e.g. social interactions costing lots of energy)], impact on daily life (restricted life domains) and presumed causes of fatigue; (ii) experiences and needs regarding discussion and treatment of fatigue during routine nephrology care: discussion with treating physician (frequency, reasons not to discuss), advice/treatment(s) given, treatment-effectiveness, and tips for fellow patients; and (iii) experiences and needs for (social) support. Finally, the following demographic and clinical characteristics were collected via the survey: age, gender, marital status, educational level, current working status, ethnocultural background, CKD population (CKD, dialysis, KTx), comorbidities [diabetes mellitus, chronic obstructive pulmonary disease (COPD), heart failure], and other symptoms (sleep problems, depressive feelings).

### Analysis

Descriptive statistics were used to describe patients’ characteristics and to summarize results of close-ended questions about fatigue for the total population and also stratified by CKD population (CKD, dialysis, after KTx), gender (men, women), and age (<50, 50–70, ≥70 years) [16]. To avoid missing data, this survey only used mandatory fields. Answers to open-ended questions were categorized into groups and ranked. All analysis were performed using SPSS version 29.0 and R version 4.3.1.

## RESULTS

### Patient characteristics

In Table 1, patient characteristics (*n* = 414) are presented after removing duplicate surveys (*n* = 7). Participants had a mean age of 58.4 [standard deviation (SD) 13.6] years, and most patients were women (57.5%), highly educated (44.9%), currently not working (69.1%), and married/cohabiting (70.8%). In total, 34.8% were patients with CKD (*n* = 144), 9.4% received dialysis (*n* = 39), and 55.8% received a KTx (*n* = 231). Comorbidities were common, namely diabetes (18.1%), COPD/long emphysema (5.8%), heart failure (11.8%), sleeping problems (30.2%), and depressive feelings (16.2%).

**Table 1:** Baseline characteristics of the different CKD populations.

	Total population	CKD	Receiving dialysis	After KTx^a^
Characteristics	*n* = 414	*n* = 144 (34.8%)	*n* = 39 (9.3%)	*n =* 231 (55.8%)
Gender				
man, *n* (%)	176 (42.5%)	55 (38.2%)	17 (43.6%)	104 (45.0%)
woman, *n* (%)	238 (57.5%)	89 (61.8%)	22 (56.4%)	127 (55.0%)
Age (years)	58.4 (13.6)	57.9 (14.3)	61.9 (15.4)	58.2 (12.8)
<50 years, *n* (%)	106 (25.6%)	45 (31.3%)	6 (15.4%)	55 (23.8%)
50–70 years, *n* (%)	213 (51.4%)	66 (45.8%)	21 (53.8%)	126 (54.5%)
≥70 years, *n* (%)	95 (22.9%)	33 (22.9%)	12 (30.8%)	50 (21.6%)
Ethnocultural background, Dutch, *n* (%)^b^	393 (94.9%)	139 (96.5%)	38 (97.4%)	216 (93.5%)
Educational level, high, *n* (%)	186 (44.9%)	60 (41.7%)	15 (38.5%)	111 (48.1%)
Marital status, *n* (%)^c^				
married/cohabiting	293 (70.8%)	111 (77.1%)	25 (64.1%)	157 (68.0%)
married/not cohabiting	15 (3.6%)	4 (2.8%)	2 (5.1%)	9 (3.9%)
residing with family	9 (2.2%)	3 (2.1%)	1 (2.6%)	5 (2.2%)
single/living alone	96 (23.2%)	26 (18.1%)	11 (28.2%)	59 (25.5%)
Current working status, *n* (%)				
full-time	33 (8.0%)	16 (11.1%)	3 (7.7%)	14 (6.1%)
part-time, hours a week^d^	95 (22.9%) 20.7 (8.3)	36 (25.0%) 19.6 (9.2)	3 (7.7%) 10.3 (8.5)	56 (24.2%) 22.0 (7.2)
not working^e^	286 (69.1%)	92 (63.9%)	33 (84.6%)	161 (69.7%)
Comorbidities/other symptoms, *n* (%)				
diabetes mellitus	75 (18.1%)	14 (9.7%)	8 (20.5%)	53 (22.9%)
COPD/emphysema	24 (5.8%)	11 (7.6%)	4 (10.3%)	9 (3.9%)
heart failure	49 (11.8%)	22 (15.3%)	7 (17.9%)	20 (8.7%)
depressive feelings	67 (16.2%)	24 (16.7%)	4 (10.3%)	39 (16.9%)
sleep problems	125 (30.2%)	43 (29.9%)	13 (33.3%)	69 (29.9%)

Normally distributed continuous values are presented as mean ± SD and categorical variables as numbers (percentages).

a KT, kidney transplantation.

b *n* = 3 (0.7%) indicated that they did not want to state this.

c *n* = 1 (0.2%) opted otherwise but did not complete the open question.

d *n* = 6 (1.4%) did not indicate how many hours they worked.

e Reasons for currently not working include retirement, declared incapacitated for work, and on sick leave.

### Experiences with and presumed causes of fatigue

**Figure 1:**
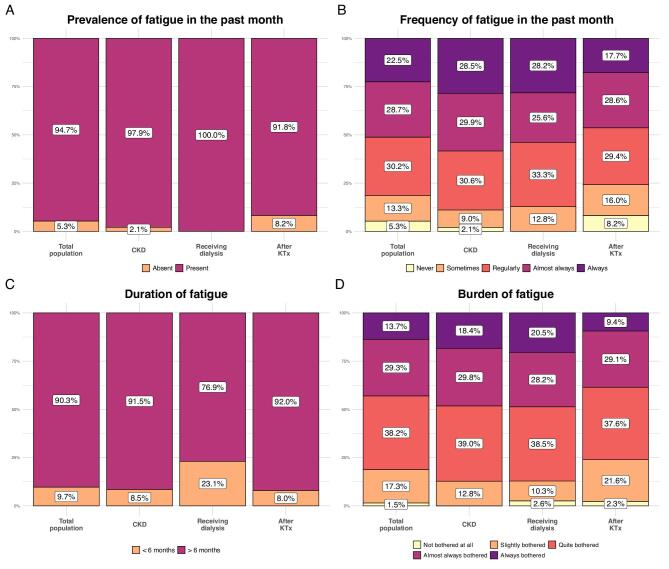
(**A**) Prevalence of fatigue in the past month. (**B**) Frequency of fatigue in the past month. (**C**) Duration of fatigue. (**D**) Burden of fatigue. The percentages are shown for the total population and for the different CKD populations (patients with CKD, receiving dialysis, after KTx).

Almost all patients (94.7%) experienced fatigue in the previous month and for more than six (90.3%), of whom 51.2% experienced fatigue (almost) all the time. Most patients (81.2.%) reported a high burden of fatigue, and 86.3% ranked fatigue in the top three most burdensome symptoms (Fig. 1). Patients noticed fatigue on multiple levels: they were physically tired (81.4%), mentally (68.2%), socially (50.1%), and/or emotionally (48.1%). Most restrictions in daily life were experienced during house-related tasks (77.9%), social activities (69.0%), relationship/family life (40.7%), and education/work (36.1%) (Fig. S1).

All CKD populations (CKD, dialysis, KTx) experienced fatigue often (97.9%, 100%, and 91.8%, respectively), experienced a high burden (87.2%, 87.2%, and 76.1%), and considered fatigue in the top three most burdensome symptoms (87.2%, 79.5%, and 86.9%) (Fig. 1). Additionally, patients in all CKD populations felt physically, mentally, socially, and/or emotionally tired, but more patients with CKD noticed social and emotional fatigue. Moreover, patients receiving dialysis felt less constrained in education/work than the other CKD populations (Fig. S1). More women experienced a higher fatigue burden, noticed more emotional and social fatigue, and were more often restricted in household-related tasks and social activities than men. The fatigue burden was slightly decreasing in the oldest age groups, while more younger patients noticed being mentally, socially, and emotionally tired, resulting in more restrictions in social activities, education/work, and relationship/family life (Figs. S2 and S3).

The most commonly presumed causes of patients’ fatigue could be divided into the following six themes, ranked by how often they were mentioned (Table S1): (i) physiological factors [e.g. kidney disease, other disease(s), physical impairment], (ii) treatment factors (e.g. medication side-effects, kidney transplantation, dialysis treatment), (iii) psychological factors (e.g. exceeding personal boundaries, stress, mental problems, worrying), (iv) lifestyle factors (e.g. energy imbalance, workload, poor physical condition), (v) others (e.g. sleep problems/deprivation, unspecified illness, lack of energy), and (vi) demographic factors (older age, demanding work). Some patients (*n* = 33) had no idea what caused their fatigue.

### Experiences and needs for discussing and treating fatigue

**Figure 2:**
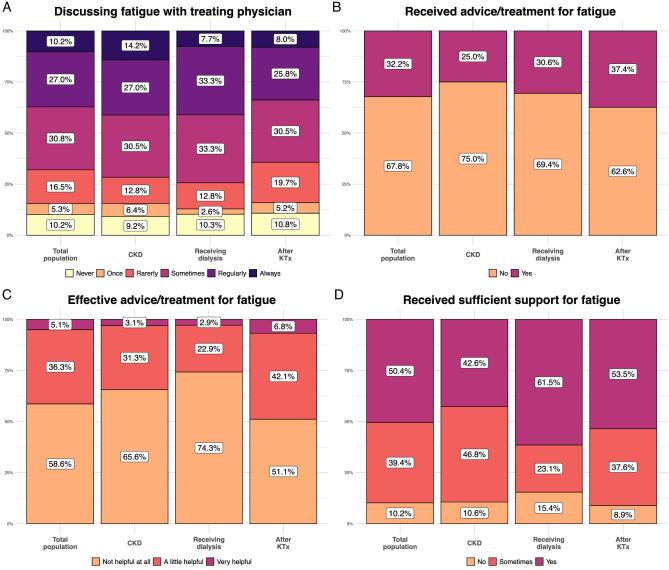
(**A**) Frequency of patients who discussed fatigue with treating physician. (**B**) Frequency of patients who received advice/treatment for fatigue. (**C**) Reported effectiveness of advice/treatment. (**D**) Perceived adequacy of current support. The percentages are shown for the total population and for the different CKD populations (patients with CKD, receiving dialysis, after KTx).

Most patients discussed fatigue at least once with their treating physician (89.8%), but (67.8%) did not receive any advice/treatment(s). Some patients (32.1%) discussed fatigue rarely or never, with the most common reasons being that patients assumed fatigue was simply part of their CKD, discussing fatigue would not change anything, and physicians’ lack of attention and efforts to discuss and/or treat fatigue. Furthermore, more patients after KTx (35.7%) rarely or never discussed fatigue than patients with CKD (28.4%) and receiving dialysis (25.6%), but patients after KTx received more often advice/treatment(s) for fatigue than the other CKD populations (37.4% vs. 25.0% vs. 30.6%, respectively) (Fig. 2). No apparent differences were found between gender and age groups (Fig. S4).

Advice and treatments that patients received from professionals included: (i) lifestyle adjustments (e.g. sufficient physical activity, taking sufficient rest, pacing oneself in daily living); (ii) medical care (e.g. erythropoietin, medication adjustment including lowering medication dose, sleep medication); (iii) paramedical care (e.g. physical therapy, consultations with medical social work, revalidation therapist); and (iv) regulation of vitamins and minerals (iron, vitamins D and B). Tips provided by patients for fellow patients included: (i) lifestyle-related tips (e.g. taking sufficient rest, sufficient physical activity, ensuring energy distribution during the day), (ii) other (mindfulness, meditation, acupuncture, other supplements), (iii) regulation of vitamins and minerals (vitamin supplements, magnesium, vitamin D3, iron), (iv) paramedical care (ergotherapy, remedial therapy, physical therapy), and (v) medical care (sleep medication, adjusting medication usage, maintaining haemoglobin levels). More than half of the patients (58.6%) believed that the professionals’ advice/treatment(s) did not help at all, 36.3% believed it helped a little, and only a few believed it helped a lot to feel less tired (5.1%). Furthermore, less patients after KTx reported that advice/treatment(s) did not work than patients with CKD and receiving dialysis (51.1% vs 65.6% vs. 74.3%, respectively). No apparent gender and age differences were detected (Figs. 2 and S4).

### Support experiences and needs

Overall, 49.6% of patients reported receiving insufficient social support for their fatigue. More patients with CKD reported insufficient social support (57.4%) than patients receiving dialysis (38.5%) and after KTx (46.5%). Moreover, more women and 18–50-year-old patients reported receiving insufficient support than men and the two older age groups (Fig. 2 and S4). The top three support-needs for all patients were: (i) acknowledgement of their debilitating symptom burdens, (ii) receiving more support from their social environment (colleagues, friends, family) and healthcare professionals, and (iii) help with household tasks. Patients also indicated that they would like to receive more information about coping strategies for dealing with (chronic) fatigue (69.8%), followed by information about treatment (68.8%), causes (36.5%), and consequences (30.9%) of fatigue. Only a few patients (12.3%) wanted to receive more information about what fatigue is. Patients with CKD were more interested in information about coping strategies than patients receiving dialysis and after KTx. Moreover, patients receiving dialysis were more interested in advice/treatment(s) than the other CKD populations. Additionally, women were more interested in advice/treatment(s), and the youngest patient group was more interested in coping strategies, advice/treatment(s), causes, and consequences of fatigue.

## DISCUSSION

This study shows that fatigue is a common and persistent symptom in patients with CKD, receiving dialysis, and after KTx. Most patients experienced a high burden and great impact on their daily life. However, many did not discuss fatigue regularly with their physician and/or perceived treatment as insufficient, resulting in unfulfilled needs. More patients with CKD, women, and younger patients reported to receive insufficient (social) support.

Consistent with our findings, previous studies found that fatigue is common, burdensome, and persistent across CKD populations [1, 17–21]. The prevalence of fatigue was higher across the different CKD populations compared to the general Dutch population [22, 23]. Moreover, previously reported fatigue prevalence rates include 78% in patients with CKD [19], 60%–97% during dialysis [20], and 86% after KTx [21]. Prevalence variations may be attributed to varying population characteristics (e.g. age, gender, comorbidities) and instruments to measure fatigue (e.g. classification scores, recall time) [19–21, 24–27]. Corresponding with previous studies, our study showed that fatigue is ranked in the top three most burdensome symptoms across different CKD populations [17, 21]. The high prevalence and burden in patients with CKD are remarkable, potentially contradicting general perceptions that fatigue is less common in this CKD population [19]. Furthermore, our findings that patients noticed fatigue on multiple levels (physical, mental, social, and/or emotional) confirm that physical fatigue is a common way to notice fatigue [5, 19] but that social, emotional, and mental fatigue is also often experienced. The latter may be partly explained by fatigue being one of the most common symptoms of patients dealing with depressive feelings [28]: a common condition in different CKD populations [29–31], including our population (16.2%). Furthermore, our patients experienced a great impact on their daily lives and (physical and mental) functioning. These findings align with previous research showing that a high symptom burden is common in patients with CKD, receiving dialysis and after KTx, which can negatively impact patients’ lives (e.g. HRQoL) [17, 32, 33].

Despite this high prevalence, burden, and negative impact of fatigue, we found that a substantial part of patients rarely discussed fatigue with their physicians and that it is only adequately managed in a small proportion of patients. Reasons for not discussing fatigue with physicians were: assumptions that fatigue was simply part of their disease, discussing fatigue would not change anything, and lack of attention and efforts from physicians to discuss and treat fatigue. These results align with previous research showing that healthcare professionals consider their communication about fatigue as poor due to limited treatment options [6]. Moreover, various studies showed that patient-reported outcomes are often underestimated, underrecognized, underdiscussed, and undertreated in routine (nephrology) care [12, 34–36]. Our study also suggests that about half of the patients received insufficient (social) support from the environment (e.g. family, friends, healthcare professionals), and want more acknowledgement of their debilitating symptom burden. These results are consistent with previous studies showing that a perceived lack of social support is common among different CKD populations [30, 37]. Moreover, higher social support levels are associated with better health outcomes in various chronic diseases (including fatigue and in CKD), suggesting that social support can be an important modifiable factor [38–41].

Regarding advice/treatments, our results confirmed findings from a previous study [20], suggesting that fatigue is often insufficiently managed. Improving our understanding of fatigue's aetiology is important to ensure adequate fatigue management. To date, the aetiology is not fully understood, but it is assumed to be multifactorial [25]. Our findings support this assumption, illustrated by the various presumed causes for fatigue: physiological, lifestyle, treatment, psychological, and demographic factors; suggesting the need for multifactorial strategies to ensure adequate fatigue support and treatment [42]. The latter is also illustrated by the advice and treatments patients received from their physician and by the tips for fellow patients, stressing the importance of pharmacological (e.g. medication adjustment including lowering medication dose) and nonpharmacological interventions (e.g. lifestyle and psychological approaches). Moreover, patients also wanted to receive more information about various topics [including treatments and coping strategies to manage (chronic) fatigue]. Future research should focus on increasing our knowledge of (modifiable) risk factors and perpetuating factors (e.g. social support, coping skills) to inform development of multifactorial treatment and support strategies. Furthermore, future research is needed to explore the impact of how advice and treatments according to healthcare professionals affects patients’ fatigue-trajectory and which treatment strategies are experienced as most effective across the different CKD populations. Further, results for the different subgroups were quite similar, although some differences should be considered. First, patients receiving dialysis experienced less barriers in education/work than the other CKD populations: results potentially explained by our dialysis patients working less than the other CKD populations [43]. Second, more younger patients noticed mental, social, and emotional fatigue than older patients and consequently experienced a greater impact on their daily lives (more limitations in education/work, social activities, relationships/family life). These results align with previous research, showing a lower mental HRQoL and higher symptom burden in younger dialysis patients than older patients [44], suggesting older patients may adapt better mentally to limitations or decreased (social) activities [45]. Although more research is needed to explore differences between patient groups (e.g. gender and cultural differences), it seems important to develop treatment and support strategies for fatigue tailored to different patient characteristics.

Taken together, patients’ high symptom burden and unmet needs underline the importance of structurally measuring the burden of fatigue in different CKD populations and ensuring that symptoms such as fatigue are discussed during routine nephrology care. Since 2018, PROMs have been implemented in all Dutch dialysis centres [13], and the first steps have been taken in the healthcare of CKD 4–5 and KTx patients [46]. However, important steps still need to be taken to embed PROMs further in routine nephrology care. Tips for structuring, discussing, and acting on PROMs from patients’ and healthcare professionals’ perspectives have been published previously [47]. Furthermore, our results suggest that patients desired multidisciplinary symptom management for fatigue, and special attention should be paid to embedding psychosocial-educational strategies to support patients in dealing with fatigue. To the best of our knowledge, current clinical nephrology guidelines do not adequately address fatigue and mainly focus on pharmacological treatments. For example, anaemia is a commonly identified risk factor and included as a treatment target in guidelines (e.g. using erythropoietin) [25, 42]. However, given anaemia's treatment ceiling effect, the multifactorial aetiology of fatigue, and patients’ need for coping strategies, it is important to broaden current fatigue management. Psychosocial interventions for fatigue have been extensively studied and recommended in other chronic diseases (e.g. rheumatoid arthritis, multiple sclerosis, cancer) [48–52]. Further research is needed to develop and evaluate the effects of multidisciplinary fatigue-management (communication and treatment) strategies and identify how to implement them into routine nephrology care.

This study has several strengths. The survey was designed in close collaboration with patients with CKD and patient-representatives, ensuring the survey adequately reflects patients’ perceptions and experiences, and enabling the development of fatigue-management strategies that fit patients’ needs. Furthermore, to the best of our knowledge, this is the first study describing experiences and needs regarding fatigue in three CKD populations with a sufficiently large sample size. However, our dialysis population could be considered relatively small, the age of patients with CKD was relatively younger, and a high percentage of patients were highly educated compared to the different Dutch CKD populations (all potentially due to using a digital survey), thereby impacting the generalizability of our results [53–55]. Moreover, data on kidney function and dialysis vintage is lacking, which could provide important context-information (e.g. affecting fatigue severity). Furthermore, selection might have occurred because women (i.e. overrepresented compared to other studies) [54], fewer patients with a KTx were employed (i.e. underrepresented compared to other studies) [55], and patients experiencing fatigue might be more inclined to complete the survey. Although our results align with previous studies, this could have led to overestimation of the prevalence and impact of fatigue. Concurrently, our patients might feel more empowered discussing their fatigue, potentially leading to an overestimation of patients discussing fatigue with their physician and/or receiving advice/treatment(s). Finally, an *ad hoc* survey was used for this fatigue-specific needs assessment across different CKD populations, and future research could focus on validating such symptom-specific needs assessment tools.

In conclusion, fatigue is a common, burdensome, and impactful symptom in different CKD populations. However, many patients do not discuss fatigue frequently with their physician and receive insufficient (social) support and treatment, which results in unfulfilled needs. Although differences between different CKD populations, genders, and ages were relatively small, it is important to be aware of differences and apply patient-centred approaches. Our results could facilitate better understandings of patients’ experiences and needs for discussion, treatment, and support for fatigue, and emphasize the importance of structurally measuring, discussing, and multidisciplinary symptom management of fatigue in routine nephrology care.

## Supplementary Material

sfaf118_Supplemental_Files

## Data Availability

The data used for this research is available upon request. Contact information: Dr Yvette Meuleman, Y.Meuleman@lumc.nl.

## PRO-GUIDE STUDY GROUP

The PRO-GUIDE study group consists of: Eline Schade van Westrum, Friedo Dekker, Marc Hemmelder, Birit Broekman, Carl Siegert, Alferso Abrahams, Ellen Hoogeveen, Coby Annema, Karen Prantl, Wanda Konijn, Marc ten Dam, Mandy Smink, Mirjam Splinter, Dirry Keurhorst, Theodôr Vogels, Willem Jan Bos, Frans van Ittersum, and Yvette Meuleman.
