# Supplementary material for: Fatigue across different chronic kidney disease populations: experiences and needs of patients
Source: Clin Kidney J. 2025 Apr 18;18(5):sfaf118. doi: 10.1093/ckj/sfaf118 (PMC12209799; doi:10.1093/ckj/sfaf118)
Supplement: sfaf118_Supplemental_Files [file sfaf118_Supplemental_Files.zip › 1475 Supplementary materials.docx]

**Supplementary materials**


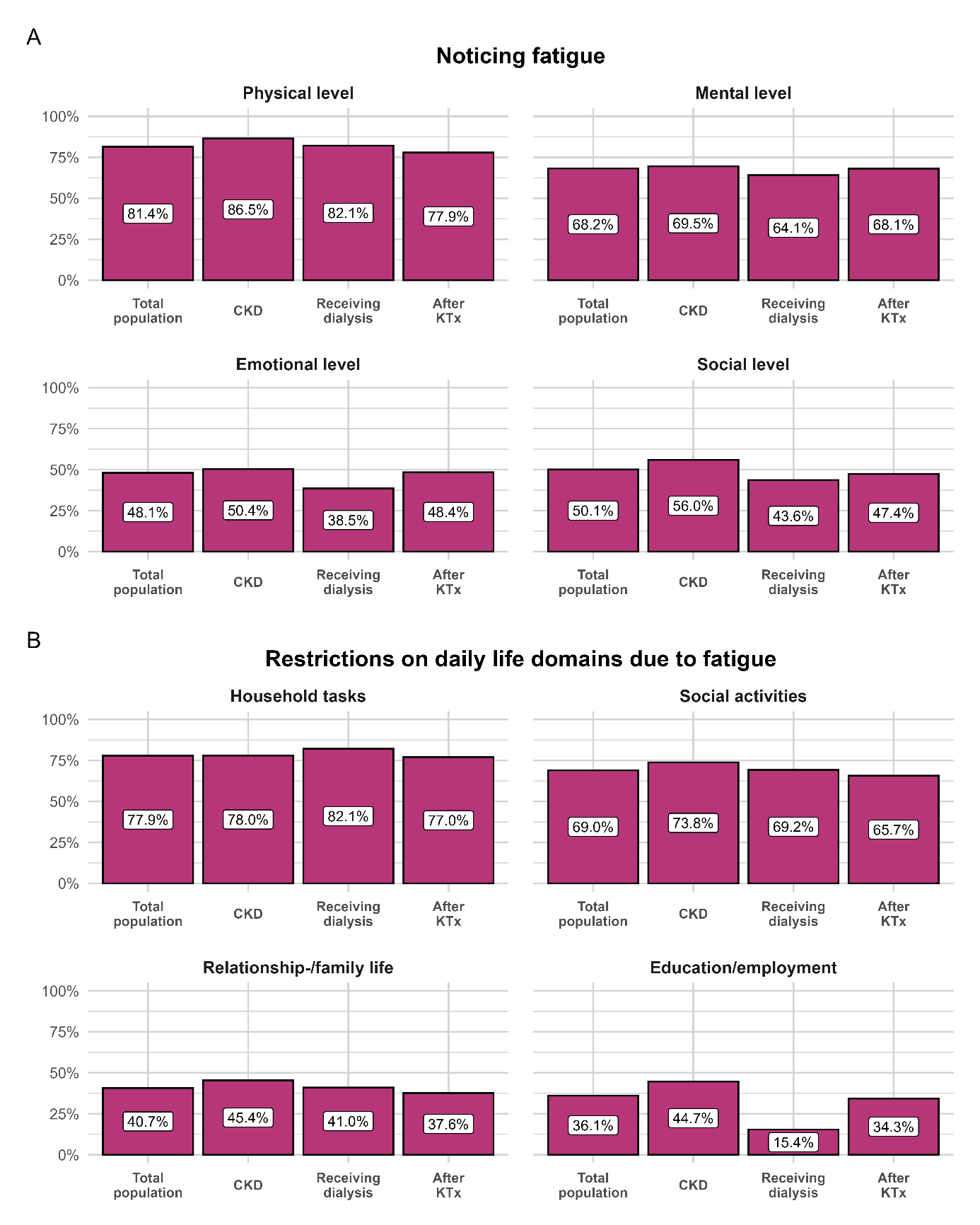


**Supplementary Figure S1: (A)** Levels on which patients noticed fatigue. On average, patients noticed their fatigue on 2.5 (SD 1.2) levels. **(B)** Experienced restrictions in life domains. On average, patients experienced 2.2 (SD 1.2) restrictions. The percentages are shown for the total population and for the different CKD-populations (patients with CKD, receiving dialysis, and after KTx).

**
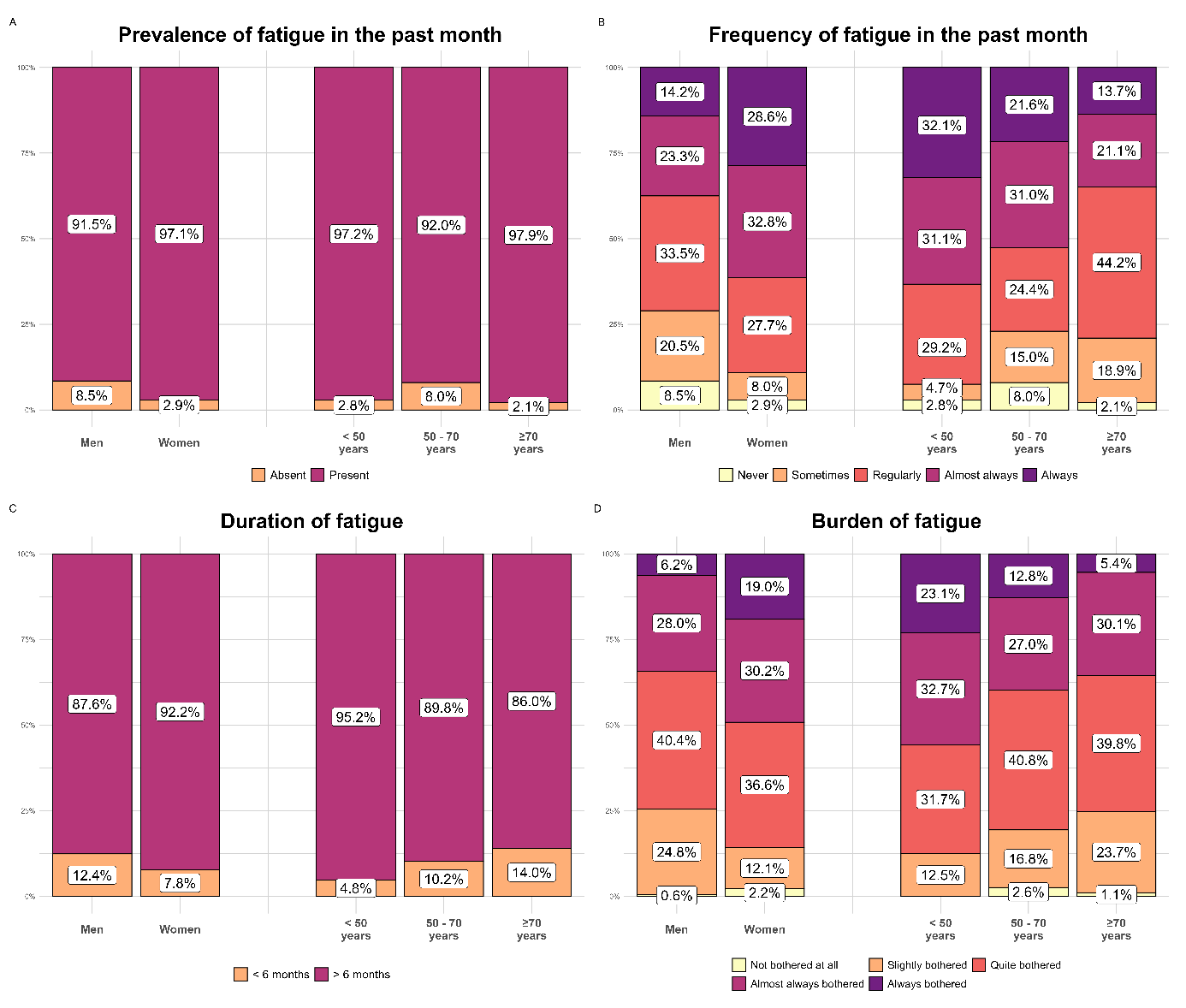
**

**Supplementary Figure S2: (A)** Prevalence of fatigue in the past month. **(B)** Frequency of fatigue in the past month. **(C)** Duration of fatigue. **(D)** Burden of fatigue. The percentages are shown for the different gender groups (men, women) and age groups (<50, 50-70, ≥70 years).

**
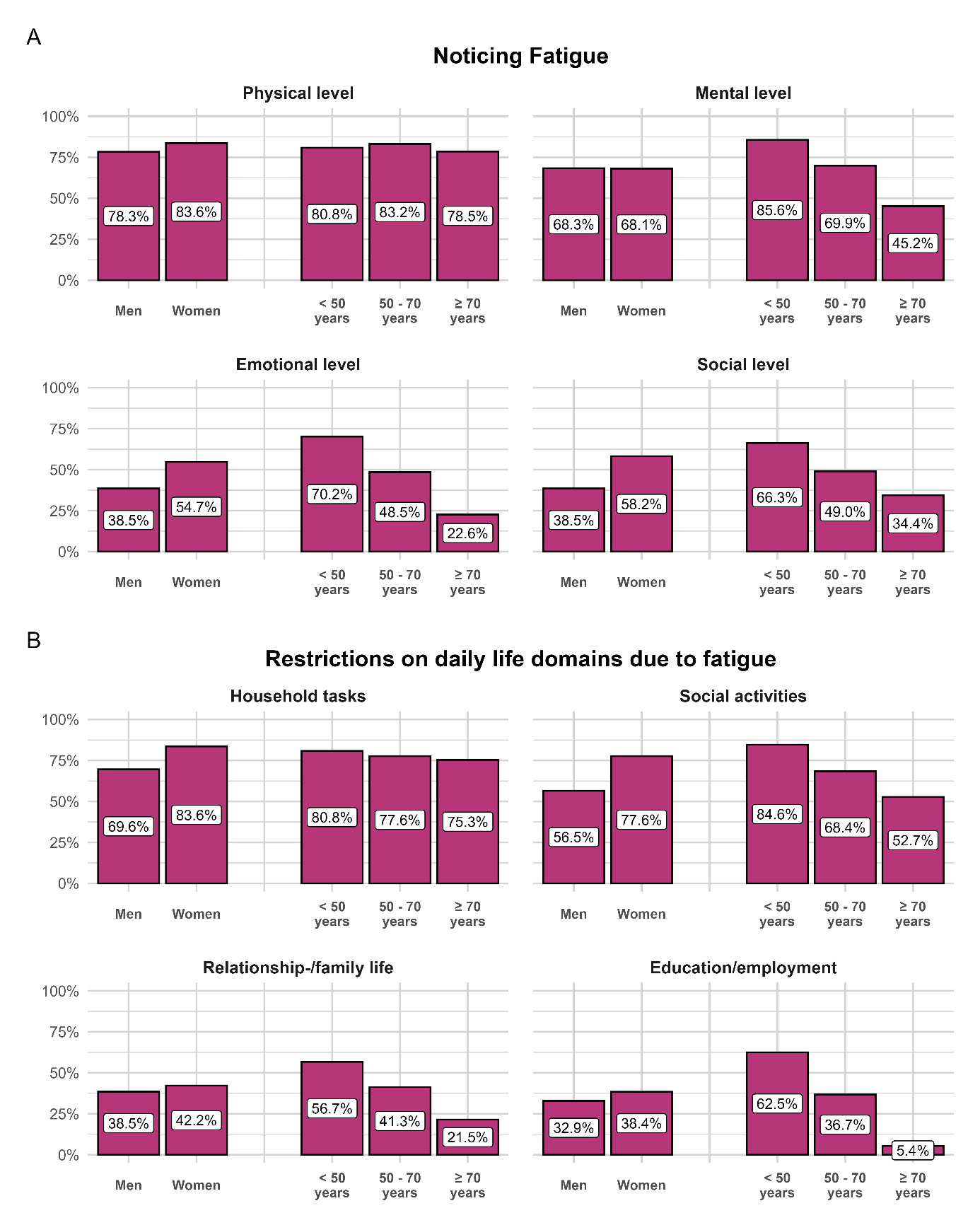
**

**Supplementary Figure S3: (A)** Levels on which patients noticed fatigue. On average, patients noticed their fatigue on 2.5 (SD 1.2) levels. **(B)** Experienced restrictions in life domains. On average, patients experienced 2.2 (SD 1.2) restrictions. The percentages are shown for the different gender groups (men, women) and age groups (<50, 50-70, ≥70 years).


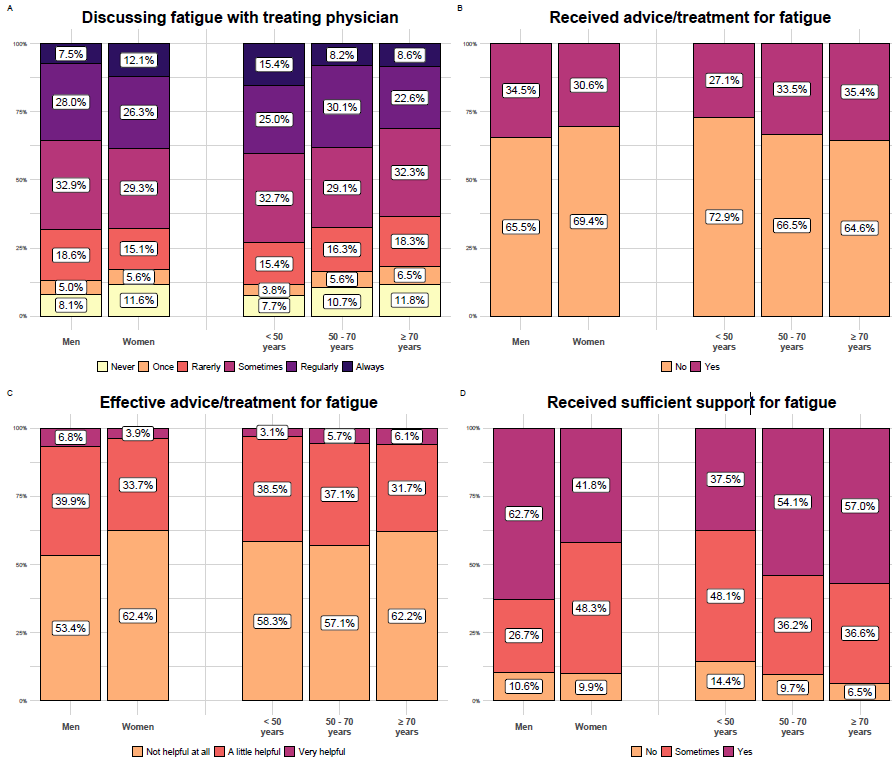


**Supplementary Figure S4: (A)** Frequency of patients who discussed fatigue with treating physician. **(B)** Frequency of patients who received advice/treatment for fatigue. **(C)** Reported effectiveness of advice/treatment **(D)** Perceived adequacy of current support. The percen­tages are shown for the different gender groups (men, women) and age groups (< 50, 50-70, ≥ 70 years).

**Supplementary Table S1:** Overview of presumed causes of fatigue according to patients^*^

| **Physiological factors** | **Treatment factors** | **Psychological factors** | **Lifestyle factors** | **Other factors** | **Demographic factors** |
| --- | --- | --- | --- | --- | --- |
| Kidney disease | Medication side effects | Exceeding personal boundaries | Energy imbalance | Sleep problems/ deprivation | Older age |
| Other disease(s) | Kidney transplantation | Stress | Workload | Unspecified illness | Demanding work |
| Physical impairment | Dialysis treatment | Mental problems | Poor physical condition | Lack of energy |  |
| Pain | Surgery | Worrying | Active lifestyle | Abnormal lab (blood) results |  |
| Uremic toxins accumulation | Cancer treatment | Depressive symptoms | Unsuitable diet | Seasonal change |  |
| Anemia |  | Anxiety |  | General health status |  |
| Physical exertion |  | Loneliness |  | Daylight saving time |  |
| Inflammation |  | Mental burden |  |  |  |
| Muscle weakness |  | Mentally processing |  |  |  |
| Micronutrients shortage |  | Psychological aspects |  |  |  |
| Infection |  | Sadness |  |  |  |
| Blood pressure |  |  |  |  |  |
| Amputated limb |  |  |  |  |  |
| Visually impaired |  |  |  |  |  |
| Protein waste |  |  |  |  |  |
| Fluid overload |  |  |  |  |  |
| Menopause |  |  |  |  |  |
| Kidney rejection |  |  |  |  |  |
| Fever |  |  |  |  |  |

* The presumed causes of patients’ fatigue are ranked according to how often they are mentioned.

**Supplementary Item S1:** Sample size calculation

The main question for the sample size calculation was question 11: the experience of fatigue in the past month. The required sample size was calculated based on the binominal distribution (present or absent):


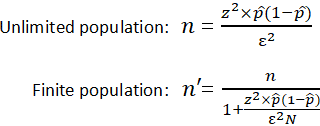


As we aimed to demonstrate a proportion rate (p̂) of 50% with a confidence of 95% (z=1.96) and a 10% margin error (ε) for the patients who did experience fatigue in the previous month, we required a sample of 97 patients. However, our aim was also to explore the differences between the CKD-populations, gender- and age groups and hence, we aimed for a sample size of at least 291 patients.

**Supplementary Item S2:** Checklist for Reporting Of Survey Studies (CROSS)

| **Section/topic** | **Item** | **Item description** | **Reported on page #** |
| --- | --- | --- | --- |
| **Title and abstract** | | |  |
| Title and abstract | 1a | State the word “survey” along with a commonly used term in title or abstract to introduce the study’s design. | 3 |
|  | 1b | Provide an informative summary in the abstract, covering background, objectives, methods, findings/results, interpretation/discussion, and conclusions. | 3 |
| **Introduction** | | |  |
| Background | 2 | Provide a background about the rationale of study, what has been previously done, and why this survey is needed. | 5 |
| Purpose/aim | 3 | Identify specific purposes, aims, goals, or objectives of the study. | 5 |
| **Methods** | | |  |
| Study design | 4 | Specify the study design in the methods section with a commonly used term (e.g., cross-sectional or longitudinal). | 6 |
|  | 5a | Describe the questionnaire (e.g., number of sections, number of questions, number and names of instruments used). | 6-7 |
| Data collection methods | 5b | Describe all questionnaire instruments that were used in the survey to measure particular concepts. Report target population, reported validity and reliability information, scoring/classification procedure, and reference links (if any). | 6-7 |
|  | 5c | Provide information on pretesting of the questionnaire, if performed (in the article or in an online supplement). Report the method of pretesting, number of times questionnaire was pre-tested, number and demographics of participants used for pretesting, and the level of similarity of demographics between pre-testing participants and sample population. | 6 |
|  | 5d | Questionnaire if possible, should be fully provided (in the article, or as appendices or as an online supplement). | 32-38 |
| Sample characteristics | 6a | Describe the study population (i.e., background, locations, eligibility criteria for participant inclusion in survey, exclusion criteria). | 6 |
|  | 6b | Describe the sampling techniques used (e.g., single stage or multistage sampling, simple random sampling, stratified sampling, cluster sampling, convenience sampling). Specify the locations of sample participants whenever clustered sampling was applied. | 6 |
|  | 6c | Provide information on sample size, along with details of sample size calculation. | 6 & 28 |
|  | 6d | Describe how representative the sample is of the study population (or target population if possible), particularly for population-based surveys. | 6 |
| Survey  administration | 7a | Provide information on modes of questionnaire administration, including the type and number of contacts, the location where the survey was conducted (e.g., outpatient room or by use of online tools, such as SurveyMonkey). | 6 |
|  | 7b | Provide information of survey’s time frame, such as periods of recruitment, exposure, and follow-up days. | 6 |
|  | 7c | Provide information on the entry process:  –>For non-web-based surveys, provide approaches to minimize human error in data entry.  –>For web-based surveys, provide approaches to prevent “multiple participation” of participants. | NA  7 |
| Study preparation | 8 | Describe any preparation process before conducting the survey (e.g., interviewers’ training process, advertising the survey). | 6 |
| Ethical considerations | 9a | Provide information on ethical approval for the survey if obtained, including informed consent, institutional review board [IRB] approval, Helsinki declaration, and good clinical practice [GCP] declaration (as appropriate). | 6 |
|  | 9b | Provide information about survey anonymity and confidentiality and describe what mechanisms were used to protect unauthorized access. | 6 |
| Statistical  analysis | 10a | Describe statistical methods and analytical approach. Report the statistical software that was used for data analysis. | 7 |
|  | 10b | Report any modification of variables used in the analysis, along with reference (if available). | NA |
|  | 10c | Report details about how missing data was handled. Include rate of missing items, missing data mechanism (i.e., missing completely at random [MCAR], missing at random [MAR] or missing not at random [MNAR]) and methods used to deal with missing data (e.g., multiple imputation). | 7 |
|  | 10d | State how non-response error was addressed. | NA |
|  | 10e | For longitudinal surveys, state how loss to follow-up was addressed. | NA |
|  | 10f | Indicate whether any methods such as weighting of items or propensity scores have been used to adjust for non-representativeness of the sample. | NA |
|  | 10g | Describe any sensitivity analysis conducted. | NA |
| **Results** | | |  |
| Respondent characteristics | 11a | Report numbers of individuals at each stage of the study. Consider using a flow diagram, if possible. |  |
|  | 11b | Provide reasons for non-participation at each stage, if possible. | NA |
|  | 11c | Report response rate, present the definition of response rate or the formula used to calculate response rate. | NA |
|  | 11d | Provide information to define how unique visitors are determined. Report number of unique visitors along with relevant proportions (e.g., view proportion, participation proportion, completion proportion). | 7 |
| Descriptive  results | 12 | Provide characteristics of study participants, as well as information on potential confounders and assessed outcomes. | 7 |
| Main findings | 13a | Give unadjusted estimates and, if applicable, confounder-adjusted estimates along with 95% confidence intervals and p-values. | NA |
|  | 13b | For multivariable analysis, provide information on the model building process, model fit statistics, and model assumptions (as appropriate). | NA |
|  | 13c | Provide details about any sensitivity analysis performed. If there are considerable amount of missing data, report sensitivity analyses comparing the results of complete cases with that of the imputed dataset (if possible). | NA |
| **Discussion** | | |  |
| Limitations | 14 | Discuss the limitations of the study, considering sources of potential biases and imprecisions, such as non-representativeness of sample, study design, important uncontrolled confounders. | 12-13 |
| Interpretations | 15 | Give a cautious overall interpretation of results, based on potential biases and imprecisions and suggest areas for future research. | 13 |
| Generalizability | 16 | Discuss the external validity of the results. | 10-12 |
| **Other sections** | | |  |
| Role of funding source | 17 | State whether any funding organization has had any roles in the survey’s design, implementation, and analysis. | 14 |
| Conflict of interest | 18 | Declare any potential conflict of interest. | 14 |
| Acknowledgements | 19 | Provide names of organizations/persons that are acknowledged along with their contribution to the research. | 13 |

**Supplementary Item S3:** Translated survey

**Survey about fatigue**

We would like to know more about your experiences with fatigue.

For each question, please tick the box that best reflects your opinion.

1. Are you *(1 answer possible)*:

- Kidney patient with chronic kidney disease
- Kidney patient receiving home dialysis
- Kidney patient receiving dialysis in a dialysis center/hospital
- Kidney patient with a kidney transplant *(date of transplantation: ......../........./............)*
- Kidney donor *(date of surgery: ......../........./............)*
- Other, namely: ...........................................................

1. What is your age: ................ years old
2. Are you a:

- Man
- Woman
- Other, namely: ...........................................................
- I would rather not say

1. Are you currently:

- Single
- Married/partnership (cohabiting with partner)
- Married/in partnership (not cohabiting with partner)
- Other, namely: ...........................................................

1. Do you have children?

- Yes, children residing in the same household
- Yes, children living somewhere else
- No

1. In which country were you born?

- …………………………………………..………………………………………………
- I would rather not say

1. What country were your biological parents born in?

- …………………………………………..………………………………………………
- I would rather not say

1. What is the highest education you completed?

- Primary education (primary school)
- Secondary education (mavo/vmbo)
- Higher secondary education (havo/vwo)
- Vocational education (MBO)
- Higher professional education (HBO)
- Higher education (university)
- Other, namely: …………………………………………………………………………………………

1. Are you currently working?

- No, because: ....................................................................................................................
- Yes, part-time (..... hours per week)
- Yes, full-time

1. Do you have any of the following conditions or symptoms? *(multiple answers possible)*

- Diabetes
- COPD/long emphysema
- Heart failure
- Depressive feelings
- Sleeping problems
- None of the above

1. Have you experienced fatigue in the past month?
   - Never *(continue to question 26)*
   - Sometimes
   - Regular
   - Almost always
   - All the time
2. How long have you been experiencing fatigue?

- Less than 6 months
- More than 6 months

1. When do you feel most tired? *(multiple answers possible)*
   - After physical exertion
   - After mental exertion
   - In the morning
   - In the evening
   - Other, namely: ................................................................................................................
2. How do you notice that you are tired? *(multiple answers possible)*

- Physical level (e.g. you have a headache or less strength)
- Mental level (e.g. you may not be able to concentrate or remember things as well)
- Emotional level (e.g. you are easily upset or irritated)
- Social level (e.g. dealing with people costs you a lot of energy)
- Other, namely: ................................................................................................................

1. Presumed causes for your fatigue: …………………………………………………………. ………………………………………………………………………………………………………………………………………….………………………………………………………………………………………………………………………………………….…………………………………………………………………………………………………………………………………………………………………………………………………………………………………………………………………………………………………………………………………………………………………………………………………………
2. How much does your fatigue bother you?

- Not at all
- A little bit
- Some what
- Quite a bit
- Very much

1. Of all the symptoms that you experience, fatigue is one of the three most burdensome symptoms.

- Not applicable, because I do not experience any other symptoms
- Disagree
- Agree

1. To what extent is your daily life hampered by your fatigue? *(multiple answers possible)*

- You do not experience any restrictions due to your fatigue
- You are restricted in chores at home (e.g. housework or chores)
- You are restricted in your social activities (e.g. visiting friends or family)
- You are restricted in your education and/or work
- You are restricted in your relationship and/or family life
- You are restricted in other life domains, namely: ……………………………………................................................................................ …………………………………………………………………………………………

…………………………………………………………………………………………

…………………………………………………………………………………………

1. Do you discuss your fatigue with your treating physician?

Never *(continue to 20)*

Only once *(continue to 21)*

Rarely *(continue to 21)*

Sometimes *(continue to 21)*

Regularly *(continue to 21)*

Always *(continue to 21)*

1. If not, what is the main reason for not discussing your fatigue with your physician? *(continue to 23)*

………………………………………………………………………………………………………………………………………………………………………………………………………………………………………………………………………………………………………………………………………………………………………………………………………………………………………………………………………………………………

1. Has your physician prescribed treatments and/or given advice for your fatigue?

- No
- Yes, namely: ………………………………………………………………………………………….……………………..………………………………………………………………………………………………………………………………………………………………..

1. If so, to what extent does the treatment and/or advice help you to feel less tired?

- It does not help
- It helps a little
- It helps a lot

1. People try all kinds of things to feel less tired. Are there things that have helped you to feel less tired and that you would like to give as a tip to other people?

………………………………………………………………………………………………………………………………………………………………………………………………………………………………………………………………………………………………………………………………………………………………………………………………

………………………………………………………………………………………………

1. Do you experience sufficient support in dealing with your fatigue from your immediate environment (partner, family, friends, etc.)?

- No
- Sometimes
- Yes

1. What support are you not receiving, but would you like to receive? From whom would you like to receive this support (partner, family, friends, healthcare professionals, etc.)?

………………………………………………………………………………………………………………………………………………………………………………………………………………………………………………………………………………………………………………………………………………………………………………………………

1. What could the Dutch Kidney Patients Organisation do to support you (or other people) in dealing with fatigue? *(multiple answers possible)*

- Provide information *(see question 27 for topics)*
- Organise workshops *(see question 27 for topics)*
- Organise contact with fellow patients
- Increase awareness of the problem of fatigue
- Lobby for more scientific research
- Lobby for a better conversation with the physician about fatigue
- Other, namely: ……………………………………………………………………………………………………………………………………………………….……………………………………………………………………………………………………………………….

1. What topics about fatigue would you like to know more about? *(multiple answers possible)*

- What fatigue is
- Causes for fatigue
- Consequences of fatigue
- Treatments and advice on managing fatigue
- How to cope with (chronic) fatigue
- Other, namely: ………………………………………………………………………………………………………………………………………………………………………………………………………………………………………………………………………………

Do you have any final comments/suggestions/wishes/ideas/questions related to this survey?

…………………………………………………………………………………………….………

…………………………………………………………………………………………….………

…………………………………………………………………………………………….………

…………………………………………………………………………………………….………

…………………………………………………………………………………………….………

…………………………………………………………………………………………….………

…………………………………………………………………………………………….………

**Thank you very much for filling out this survey!**
