# Supplementary material for: Fatigue across different chronic kidney disease populations: experiences and needs of patients
Source: Clin Kidney J. 2025 Apr 18;18(5):sfaf118. doi: 10.1093/ckj/sfaf118 (PMC12209799; doi:10.1093/ckj/sfaf118)
Supplement: sfaf118_Supplemental_Files [file sfaf118_Supplemental_Files.zip › Figure_S4.pdf]

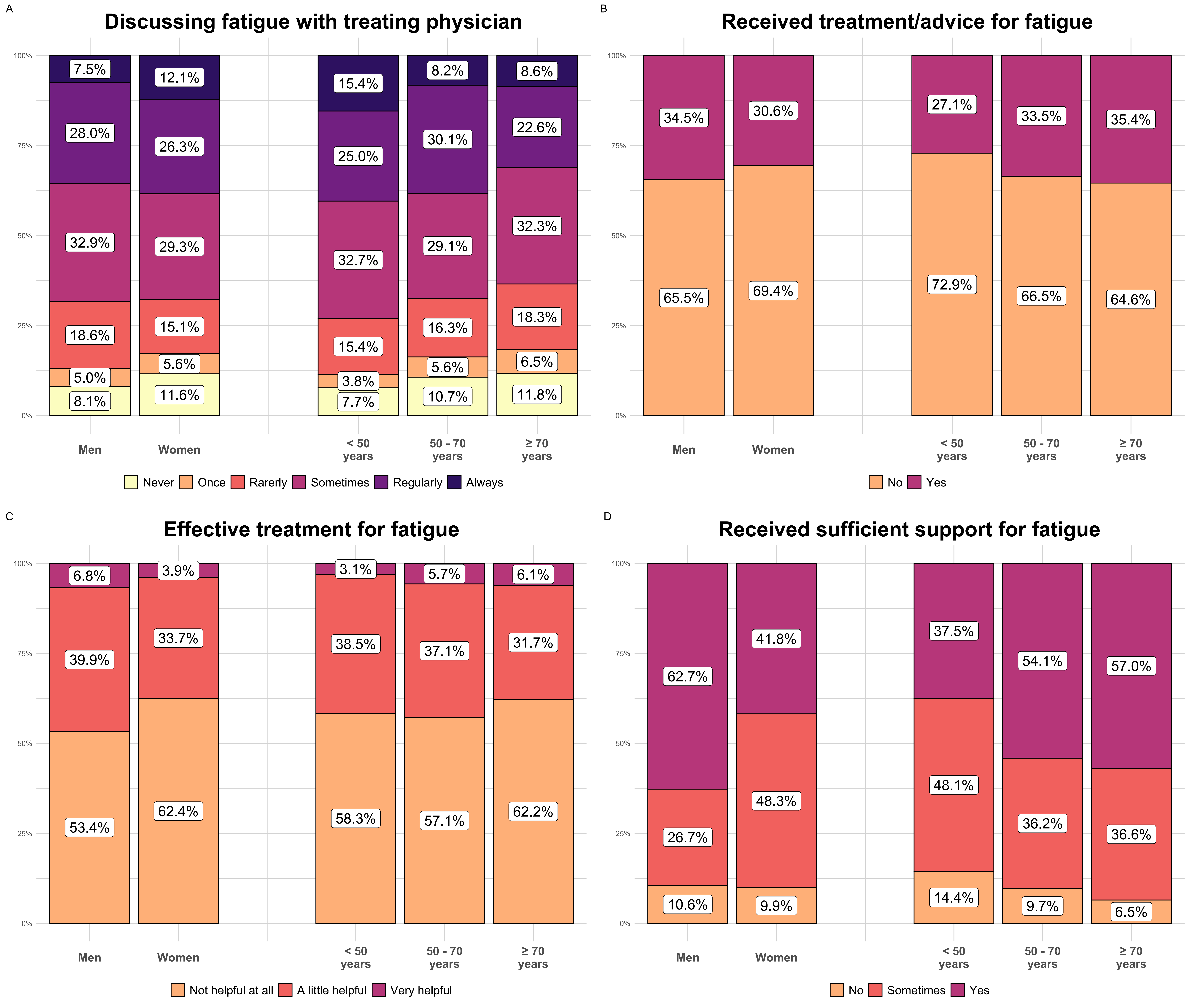

A
Discussing fatigue with treating physician
100%
75%
50%
25%
0%
7.5%
28.0%
32.9%
18.6%
5.0%
8.1%
12.1%
26.3%
29.3%
15.1%
5.6%
11.6%
15.4%
25.0%
32.7%
15.4%
3.8%
7.7%
8.2%
30.1%
29.1%
16.3%
5.6%
10.7%
8.6%
22.6%
32.3%
18.3%
6.5%
11.8%
Men
Women
< 50 years
50 - 70 years
≥ 70 years
Never
Once
Rarerly
Sometimes
Regularly
Always
B
Received treatment/advice for fatigue
100%
75%
50%
25%
0%
34.5%
65.5%
30.6%
69.4%
27.1%
72.9%
33.5%
66.5%
35.4%
64.6%
Men
Women
< 50 years
50 - 70 years
≥ 70 years
No
Yes
C
Effective treatment for fatigue
100%
75%
50%
25%
0%
6.8%
39.9%
53.4%
3.9%
33.7%
62.4%
3.1%
38.5%
58.3%
5.7%
37.1%
57.1%
6.1%
31.7%
62.2%
Men
Women
< 50 years
50 - 70 years
≥ 70 years
Not helpful at all
A little helpful
Very helpful
D
Received sufficient support for fatigue
100%
75%
50%
25%
0%
62.7%
26.7%
10.6%
41.8%
48.3%
9.9%
37.5%
48.1%
14.4%
54.1%
36.2%
9.7%
57.0%
36.6%
6.5%
Men
Women
< 50 years
50 - 70 years
≥ 70 years
No
Sometimes
Yes
