# Supplementary figures and images for: Fatigue across different chronic kidney disease populations: experiences and needs of patients
Source: Clin Kidney J. 2025 Apr 18;18(5):sfaf118. doi: 10.1093/ckj/sfaf118 (PMC12209799; doi:10.1093/ckj/sfaf118)

A

## Noticing fatigue

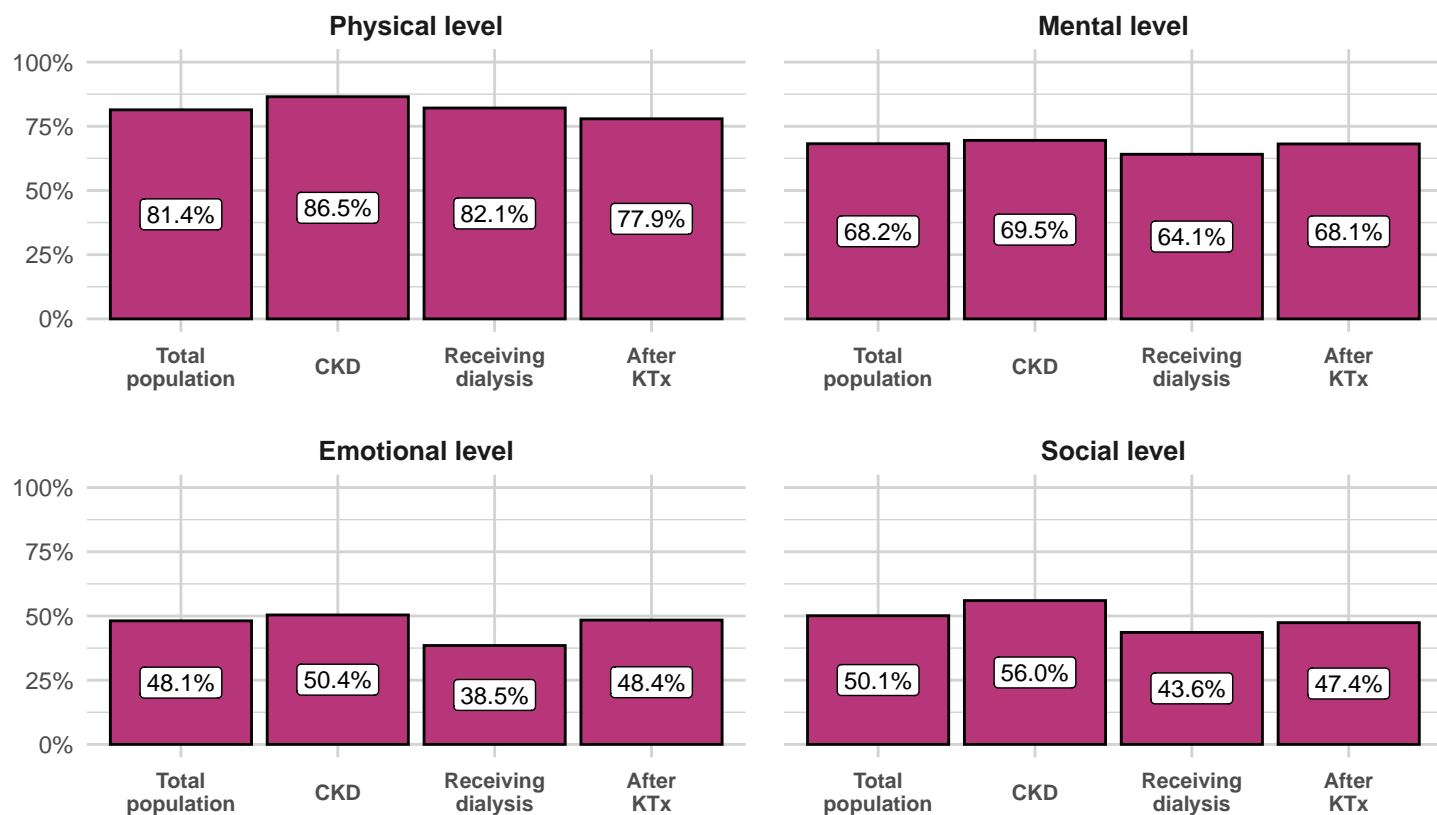

B

## Restrictions on daily life domains due to fatigue

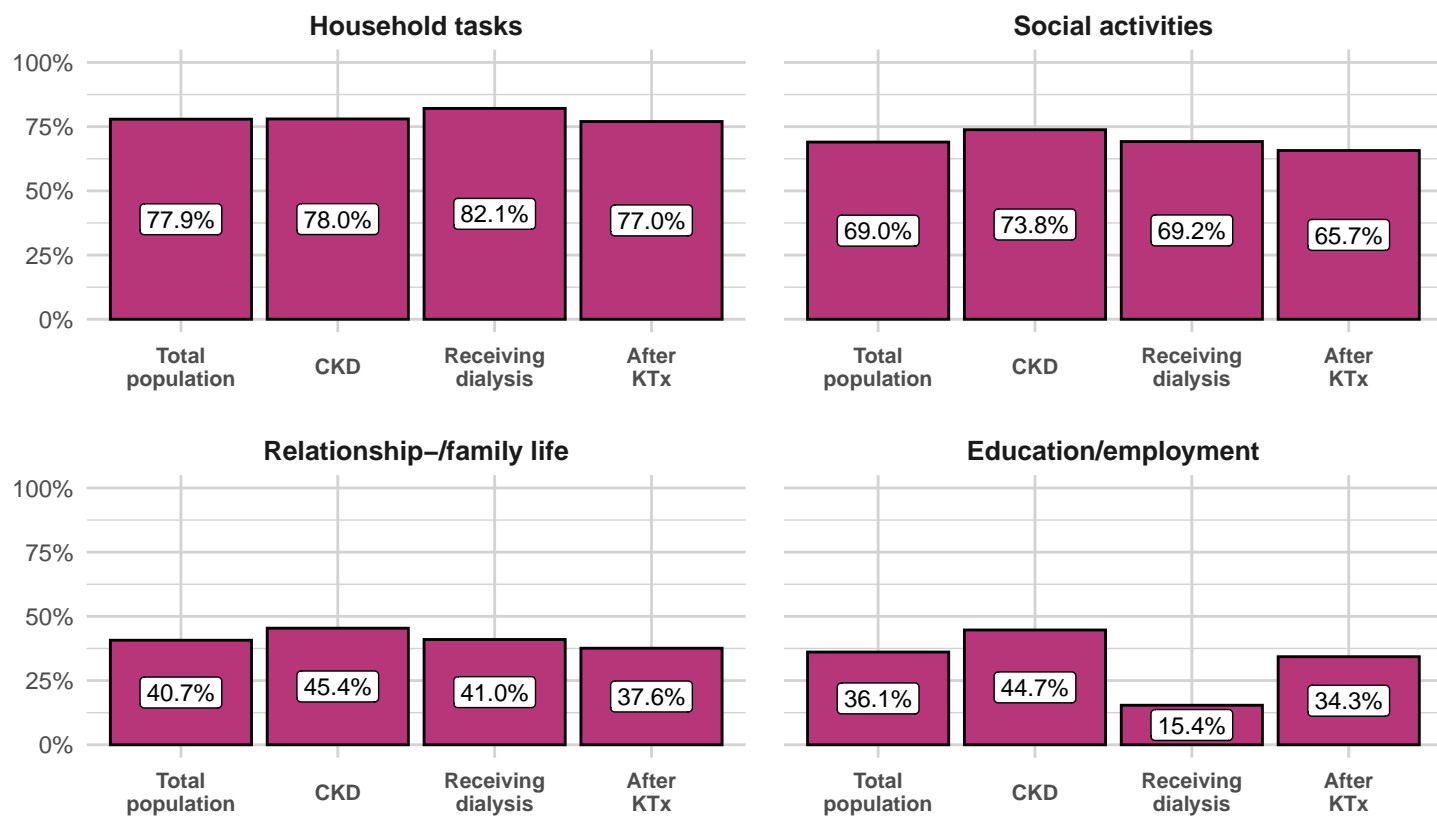

Supplement: sfaf118_Supplemental_Files [file sfaf118_Supplemental_Files.zip › Figure_S1.pdf]

A

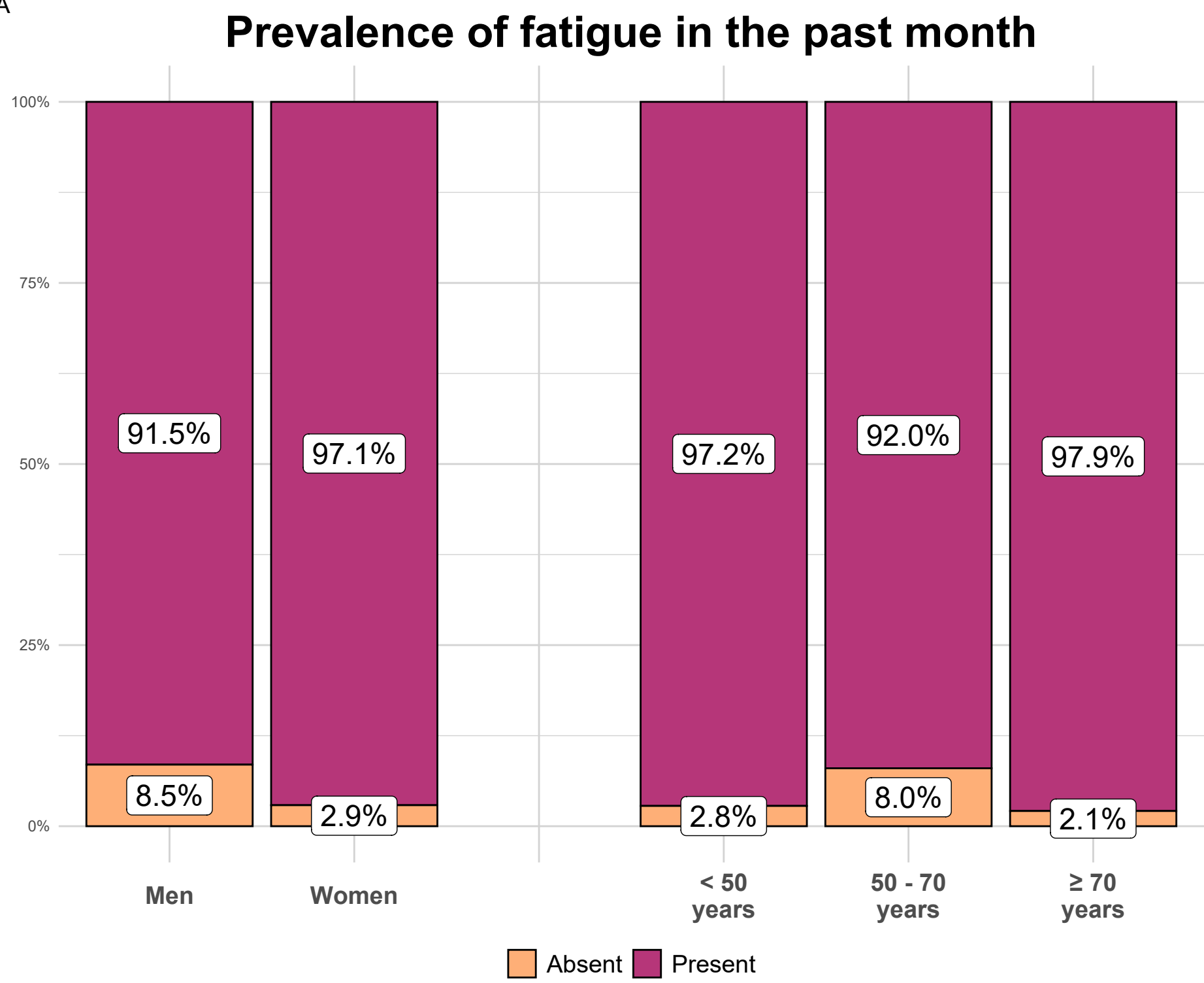

B

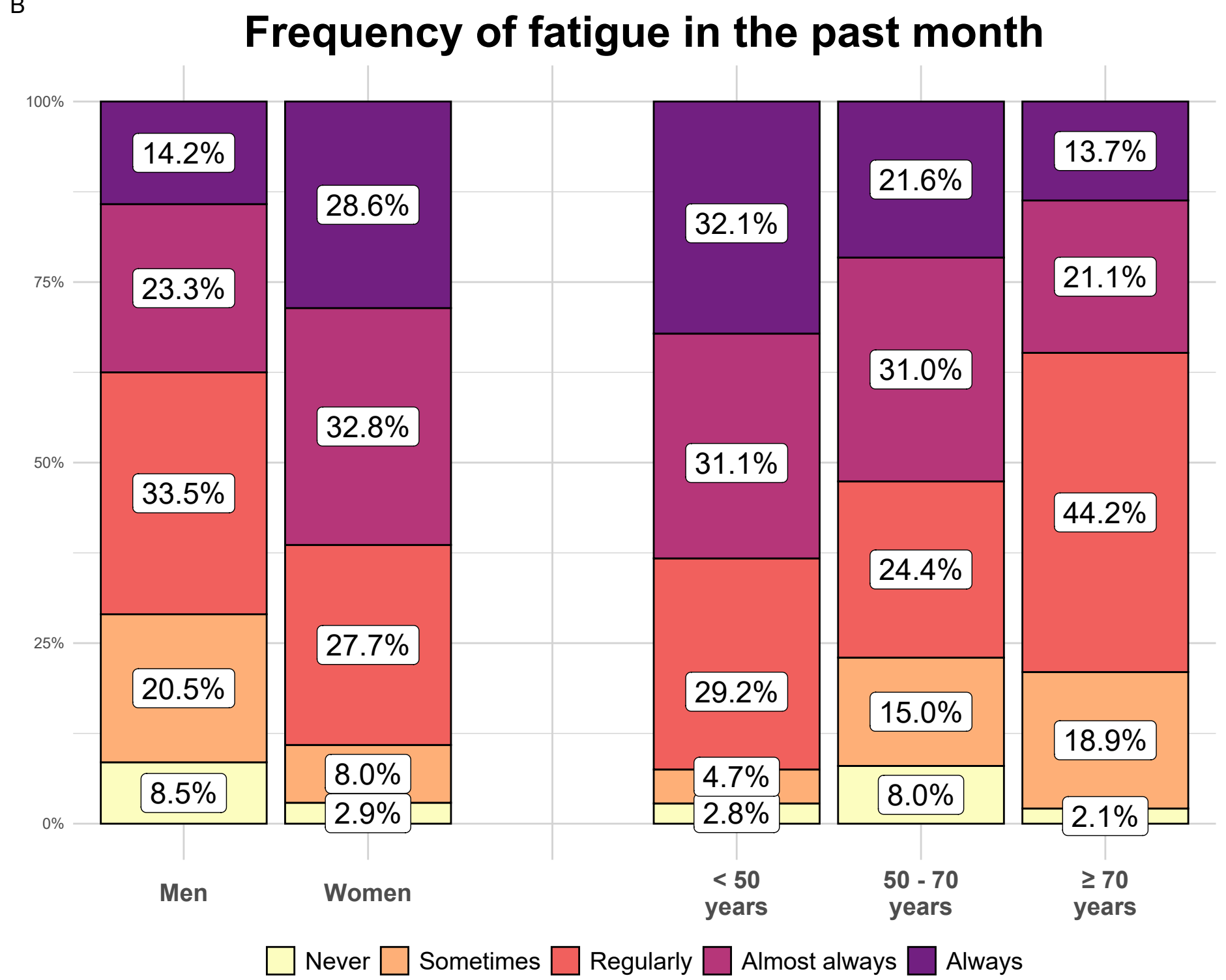

C

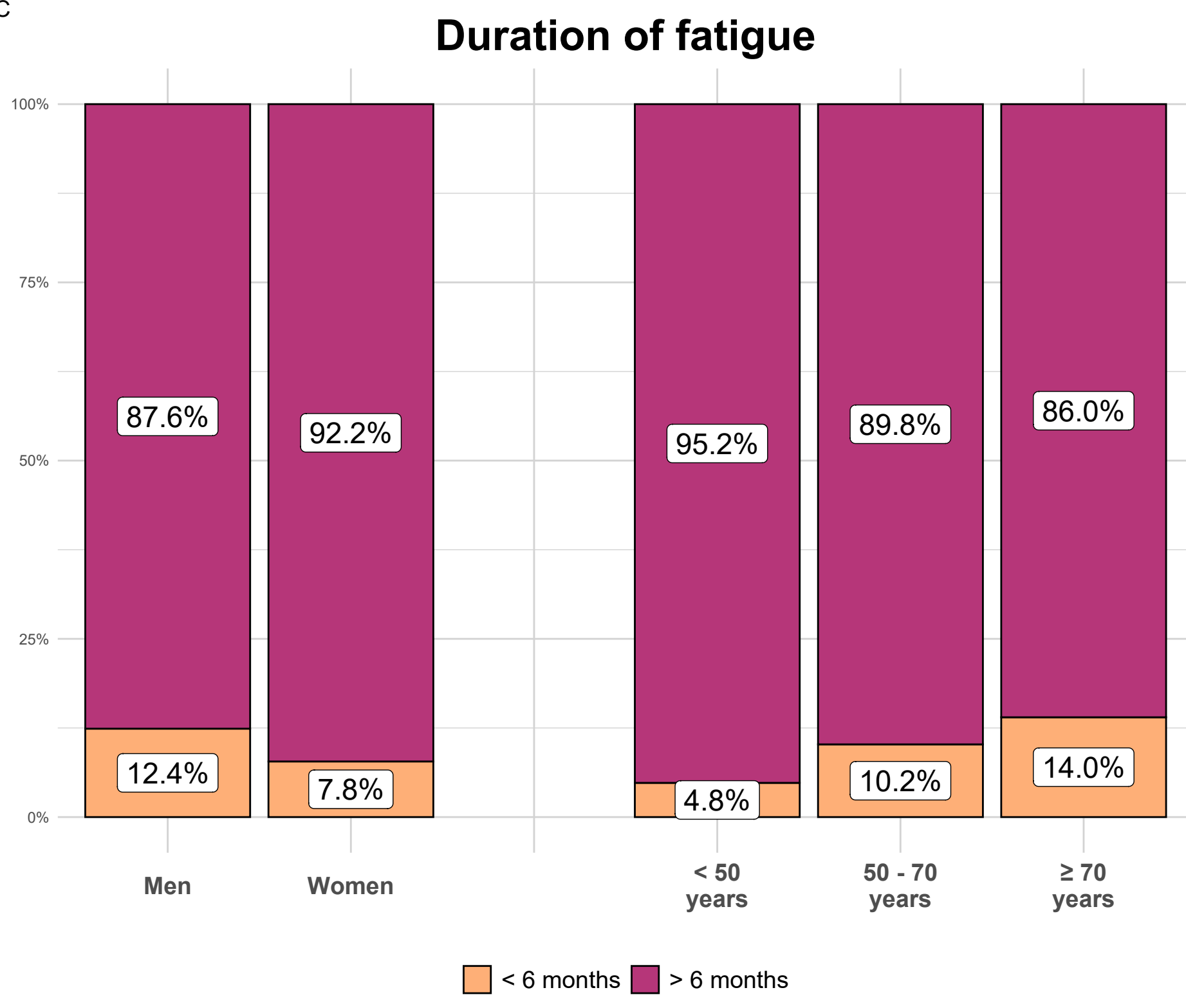

D

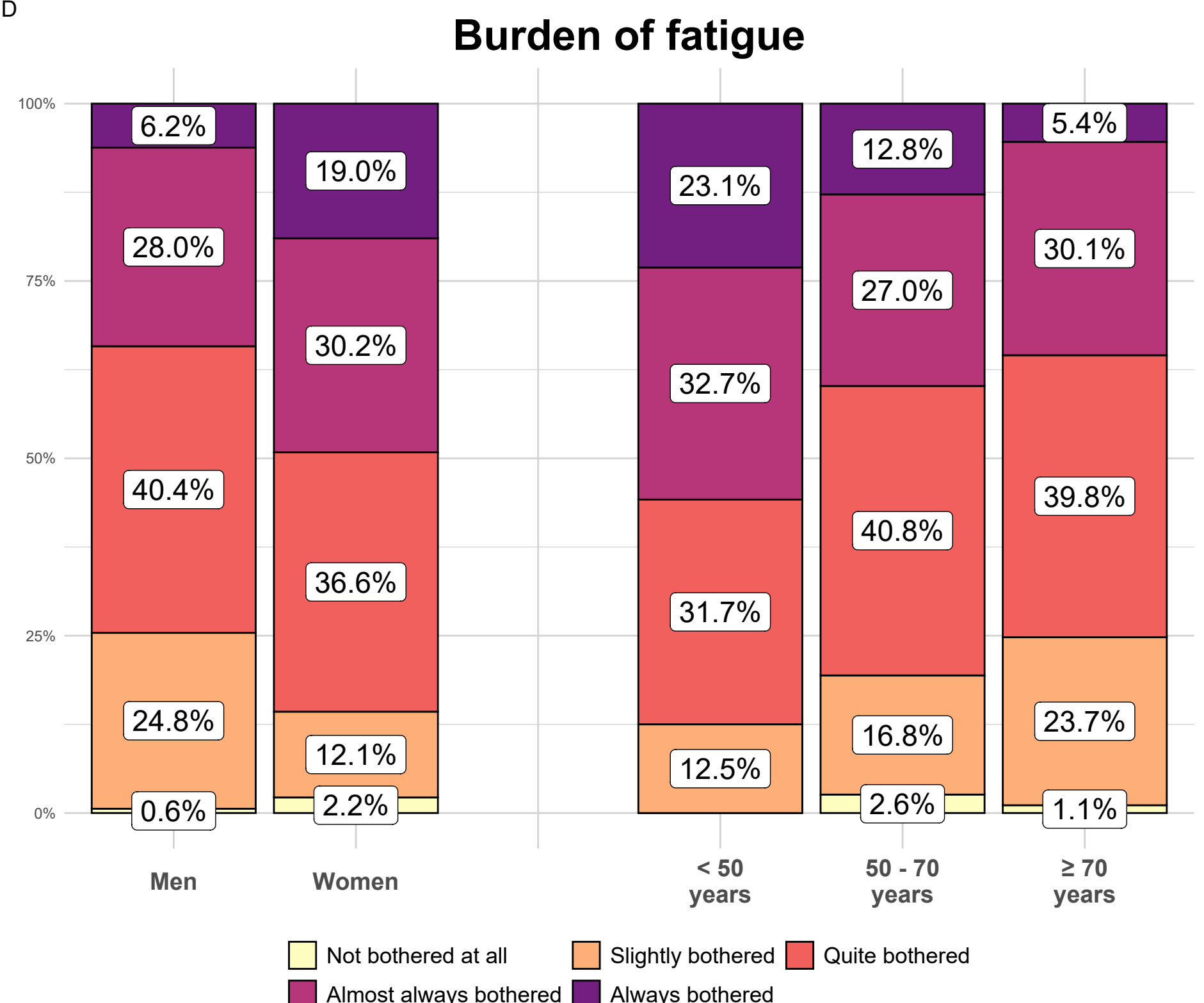

Supplement: sfaf118_Supplemental_Files [file sfaf118_Supplemental_Files.zip › Figure_S2.pdf]

A

## Noticing Fatigue

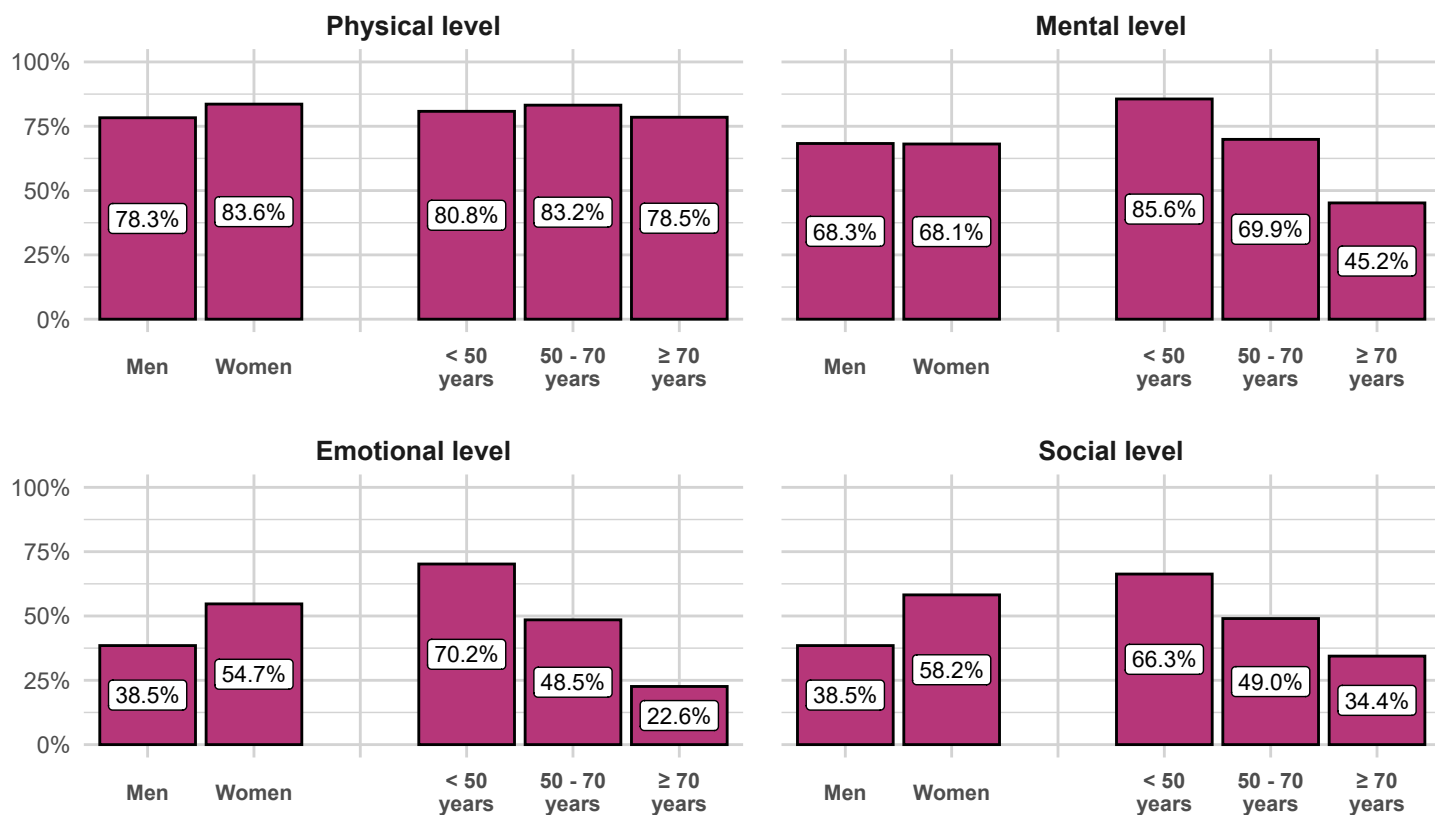

B

## Restrictions on daily life domains due to fatigue

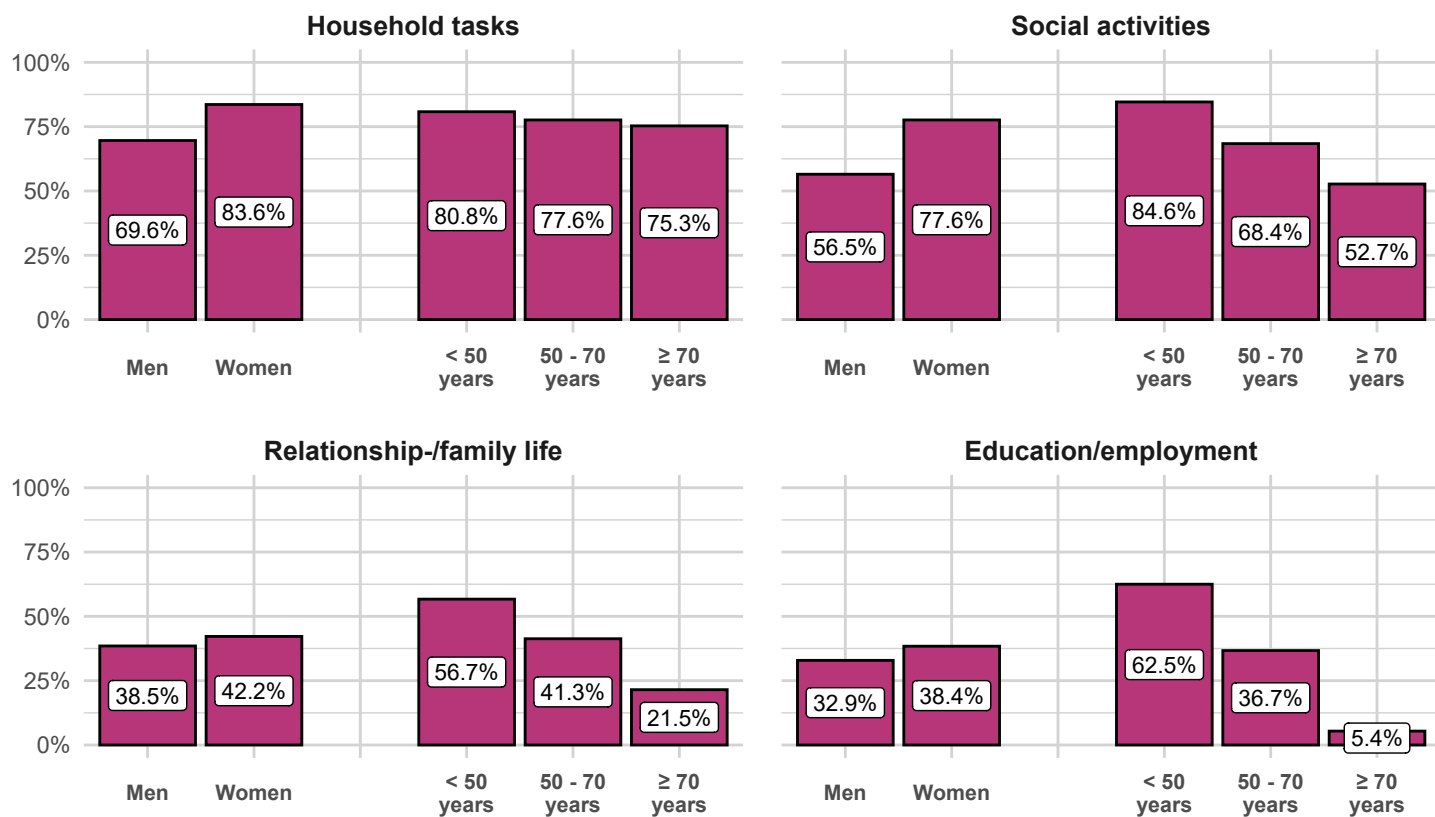

Supplement: sfaf118_Supplemental_Files [file sfaf118_Supplemental_Files.zip › Figure_S3.pdf]
